# Supplementary material for: Towards Win–Win Policies for Healthy and Sustainable Diets in Switzerland
Source: Nutrients. 2020 Sep 9;12(9):2745. doi: 10.3390/nu12092745 (PMC7551606; doi:10.3390/nu12092745)
Supplement: Supplementary file 1 [file nutrients-12-02745-s001.zip › SI PNR final/Supporting-Materials_PNR69_final.docx]

Supplementary Materials for:

Towards Win–Win Policies for Healthy and Sustainable Diets in Switzerland

# Contents

# 1 Section

# 1 Word table

# 4 Excel Tables

# SI Table S1 : Main data table, see supporting Excel file

# SI Tables S2-S3: DRFs, see supporting Excel file

# SI Table S4: Nutritional elements per diets, see supporting Excel file

# 1 figure

# Section S1. Treatment of MenuCH data

Within the MenuCH data repository food consumption is represented by 16,530 unique food descriptor codes and 3,825 unique food and recipe names, resulting in 124,190 separate entries of a survey-food combination. Composite dishes (i.e. recipes) are also expanded into the corresponding individual ingredients; therefore, in order to avoid double counting (as recommended by the MenuCH documentation), recipes were removed from the dataset (3,143 lines of data). This resulted in a database of 16,074 food descriptor codes associated with 1,519 unique food names, with a total of 121,047 lines of data. Each of these 121,047 lines of data represents a single food item that was reported during one survey for 3,860 separate surveys. This data was used as the basis of this study.

**SI table S5** Description of MenuCH surveys with respect to diets analysed

|  | Number of surveys | % of overall surveys | Number of individuals | % surveys filled by men | % surveys filled by women |
| --- | --- | --- | --- | --- | --- |
| Average | 4,144 | 100% | 2,086 | 45% | 55% |
| Vegetarian | 158 | 4% | 104 | 25% | 75% |
| Vegan | 15 | 0.4% | 9 | 33% | 67% |
| Gluten-free | 49 | 1% | 31 | 16% | 84% |
| Slimming | 178 | 4% | 143 | 30% | 70% |
| Education level 1 | 206 | 5% | 103 | 40% | 60% |
| Education level 2 | 1,934 | 47% | 973 | 40% | 60% |
| Education level 3 | 2,004 | 48% | 1,009 | 51% | 49% |

A.


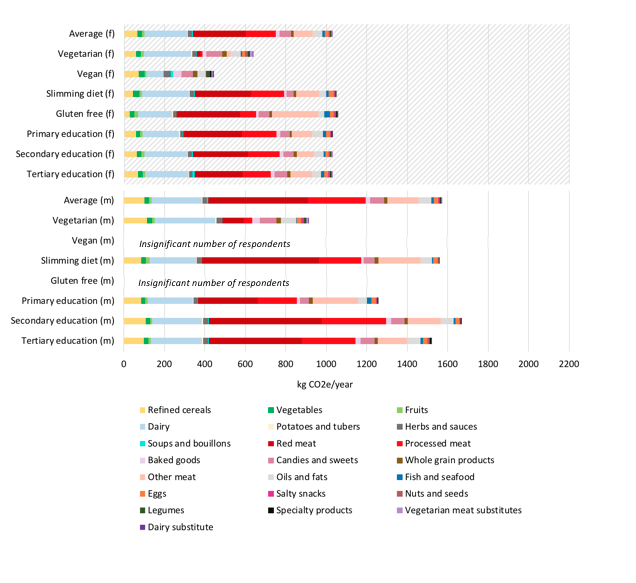


B.


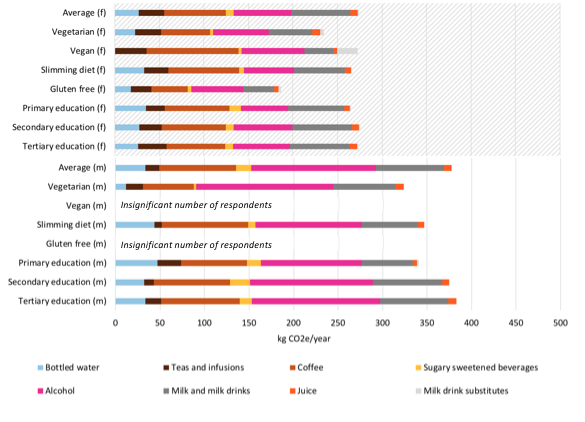


**Figure S1A-B** Total GHG emissions for Swiss diets for (A) food and (B) beverage categories without food loss and waste adjustment; note the different scale between the figures; series are ordered from most consumed to the least consumed category in the average diet. Upper charts with diagonal shading are results for women (female, f), and lower charts results for men (m).
